# Comparison of dynamic defocus curve on cataract patients implanting extended depth of focus and monofocal intraocular lens

**DOI:** 10.1186/s40662-022-00323-0

**Published:** 2023-02-01

**Authors:** Tingyi Wu, Yuexin Wang, Jiazhi Yu, Xiaotong Ren, Yuanting Li, Weiqiang Qiu, Xuemin Li

**Affiliations:** 1 Department of Ophthalmology, Peking University Third Hospital, 49 North Garden Road, Haidian District, Beijing, 100191 People’s Republic of China; 2 Beijing Key Laboratory of Restoration of Damaged Ocular Nerve, Peking University Third Hospital, Beijing, People’s Republic of China; 3 Peking University Health Science Center, Beijing, People’s Republic of China

**Keywords:** Dynamic defocus curve, Age-related cataract, Extended depth-of-focus intraocular lens, Monofocal intraocular lens

## Abstract

**Background:**

The aim of the study was to compare the dynamic defocus curve on patients post-implantation of the extended depth-of-focus (EDOF) and monofocal intraocular lens (IOL).

**Methods:**

A total of 62 age-related cataract patients receiving phacoemulsification with implantation of TECNIS Symfony (ZXR00) or monofocal IOLs were enrolled. The binocular static and dynamic defocus curves with corrected distance visual acuity were evaluated at one month postoperatively.

**Results:**

The ZXR00 group achieved significantly better intermediate (*P* = 0.044) and near (*P* = 0.017) visual acuity (VA) than the monofocal group. Two groups had similar uncorrected and corrected distance VA (*P* > 0.05, respectively). The dynamic defocus curve revealed a smoother decline from 0.0 D to − 2.0 D in the ZXR00 group. Defocused dynamic VA in the ZXR00 group was significantly better (*P* < 0.05) except at 0.0 D (*P* = 0.724) and − 0.5 D (*P* = 0.176). The area under the curve (*P* = 0.002) and corrected dynamic vision accommodation (*P* = 0.001) derived from the dynamic defocus curves were better in the ZXR00 group. A positive correlation was observed between defocused dynamic and static VA in both groups (*P* < 0.001). Multiple linear regression analysis indicated that defocused static VA and corrected dynamic vision accommodation were significant influential factors for the defocused dynamic VA from − 1.0 D to − 3.0 D (*P* < 0.05).

**Conclusions:**

The EDOF IOL provided similar distance vision, better intermediate and near vision, and a better overall dynamic defocus curve than the monofocal IOL. The dynamic defocus curve may be comprehensively applied to evaluate the all-distance dynamic visual performance post-cataract surgery.

**Supplementary Information:**

The online version contains supplementary material available at 10.1186/s40662-022-00323-0.

## Background

Age-related cataract remains a leading cause of blindness globally, and the number of cases is expected to increase steadily causing an increase in the demand for cataract surgery [1, 2]. Conventional monofocal intraocular lens (IOL) usually provides fixed focus at far or near distance, resulting in additional requirements on glasses for good near or distant vision [3]. Although multifocal IOLs liberate patients from spectacles when accomplishing near tasks, complaints such as dysphotopsias are reported [4]. The extended depth-of-focus (EDOF) IOL provides favorable distant and intermediate vision and useful near vision with a lower compromise of dysphotopsias [5, 6]. The defocus curve test is crucial for evaluating the continuous visual function at different distances after implanting IOLs, especially functional ones [7]. Compared with monofocal IOLs, EDOF IOLs demonstrate a smoother defocus curve [8]. Currently, only static optotypes are used in the defocus curve test. The test could only provide limited information on visual function because dynamic visual targets make up the majority of daily lives. The performance of continuous dynamic visual function at different distances following implantation of EDOF IOLs remains to be investigated.

The dynamic visual acuity (VA) test focuses on assessing the ability to identify dynamic optotypes at a certain distance, and may reflect real-life visual function better [9]. The performance of dynamic vision significantly affects our daily tasks such as sports and driving safety [10, 11]. Our previous study enrolled healthy subjects and showed that the defocused dynamic VA drew a similar but different curve compared with the static defocus curve, and the dynamic accommodative function derived from the dynamic defocus curve was significantly related to the defocused dynamic VA [12]. It demonstrates that the dynamic defocus curve test is a useful tool for assessing the dynamic VA under different distances. To the best of our knowledge, the defocused VA has not been evaluated on cataract patients. Therefore, this pilot study aims to apply the dynamic defocus curve test to postoperative cataract patients to evaluate the performance of continuous dynamic visual function and compare the defocused dynamic VA following implantation of EDOF and monofocal IOLs.

## Materials and methods

### Participants

The study was prospective cohort research on defocused dynamic VA in age-related cataract patients. The research was reviewed and approved by the Ethics Committee of Peking University Third Hospital (No. LM2021197) and was performed in accordance with the Declaration of Helsinki. Written informed consent was obtained from each participant. The selected IOLs were either TECNIS Symfony IOL (ZXR00, Johnson & Johnson Vision, Inc., Santa Ana, CA, USA) or one-piece acrylic monofocal aspheric IOLs [A1-UV, Eyebright Medical Technology (Beijing) Co., Ltd., Beijing, China; SZ-1, NIDEK Co., Ltd., Aichi, Japan; Aqua-Sense PAL, Aaren Scientific, Inc., Ontario, CA, USA; Aspira-aA, HumanOptics Aktiengesellschaft, Erlangen, Germany; 868UV, U.S. IOL, Inc., Lexington, KY, USA].

Inclusion criteria included continuous patients aged 50–80 years, diagnosed with binocular age-related cataracts and scheduled for bilateral cataract surgery with phacoemulsification and IOL implantation of EDOF or monofocal IOL. Patients were excluded from the study if they had high myopia (≤ − 6.00 D), high corneal astigmatism (≥ 2.00 D), history of intraocular surgery, vestibular dysfunction, congenital disorders, and underlying ocular diseases such as corneal diseases, retinopathy and glaucoma. The patients whose postoperative corrected distance VA (CDVA) of either eye worse than 0.1 logMAR were also excluded.

### Clinical evaluation

Demographic information, including name, age, sex and medical history was collected before the surgery. The preoperative ophthalmic evaluation included uncorrected distance VA (UDVA, standard logMAR VA chart), non-contact tonometry, slit lamp examination, non-mydriatic fundus photography, ocular biometry (IOL Master 700, Carl Zeiss Meditec AG, Jena, Germany), corneal topography (Pentacam, OCULUS Optikgeräte GmbH, Wetzlar, Germany), optical coherence tomography (Heidelberg Engineering GmbH, Heidelberg, Germany) and noncontact specular microscope (Nidek CEM-530, NIDEK Co., Ltd., Aichi, Japan).

Routine postoperative visits at one day and one week included the measurement of UDVA, non-contact tonometry and slit-lamp biomicroscopy. Postoperative examinations performed at 1 month ± 5 days following the cataract surgery included the measurement of UDVA and CDVA (5 m), uncorrected intermediate VA (UIVA, 80 cm), uncorrected near VA (UNVA, 40 cm), binocular static defocus curve test and dynamic defocus curve test.

### Static and dynamic defocus curve test

The binocular static defocus curve test was conducted based on CDVA. The patients wore glasses to correct the residual refractive error before the test. Additional lenses were added binocularly from + 1.0 D to − 3.0 D at a step of 0.5 D for defocusing. Patients were required to identify the optotypes on the logMAR VA chart at 5 m.

The binocular dynamic defocus curve test has been described in detail in our previous report [12]. Briefly, the examination was based on CDVA. The horizontally moving optotypes presented on a screen were generated by a self-designed program using MATLAB 2017b (The MathWorks, Inc., Natick, MA, USA). The configuration and size of the optotypes were designed according to the standard logMAR VA chart. The velocity was set at 40 degrees per second according to the previous study [12]. Patients were required to sit at 3 m in front of the screen and located their eye level at the middle of the screen. During the test, the letter E with random direction moved horizontally from the left side to the right side in the middle of the screen. The patients were asked to state the opening direction of the dynamic optotypes. We recorded the minimal size (logMAR) that the subjects could recognize, and the test was performed under different defocus statuses. Defocused lenses were added in the same way as the static defocus curve test. A spline curve was fitted to the static and dynamic defocus data [13].

The corrected dynamic vision accommodation was calculated based on the dynamic defocus curve. It was defined as the diopter range in which the patient’s dynamic VA was within the dynamic VA of 0.0 D plus 0.1 divided by the dynamic VA of 0.0 D. The elaboration can be found in our previous study [12].

### Surgical procedures

All surgeries were performed by experienced ophthalmologists (QWQ and LXM) from Peking University Third Hospital using standard phacoemulsification and implantation of IOL. Topical anesthesia was given after routine disinfection. The 3.2 mm main incision was performed at 135 degrees and the viscoelastic agent was injected into the anterior chamber afterward. The clear corneal assisted incision was performed at 45 degrees. Continuous curvilinear capsulorhexis 5–5.5 mm in diameter was conducted with capsulorhexis forceps. Balanced salt solution was injected into the lens for hydrodissection and hydrodelineation. Subsequently, coaxial phacoemulsification was applied followed by irrigation/aspiration to remove the cataractous lens. The selected IOL was inserted into the capsule through the main incision.

### Statistical analysis

Statistical analysis was operated with SPSS (version 26.0, IBM Corp., Armonk, NY, USA). The Kolmogorov-Smirnov test was applied to check the normal distribution of the data. Continuous variables were represented as means ± standard deviation (SD). Defocus curves were plotted with GraphPad Prism (version 9.0.0, GraphPad Software, San Diego, CA, USA), and the calculation of the area under the curve (AUC) using the trapezoidal rule [13] was accomplished with the same software. The comparisons of UDVA, CDVA, UIVA, UNVA, defocused dynamic VA, AUC, corrected dynamic vision accommodation and the difference value of defocused dynamic and static VA between the two groups were accomplished by the Mann-Whitney U test or independent t-test according to the normality of data. The UDVA before and after surgery were compared by the paired t-test. The correlation between the dynamic and static VA, the difference value and defocus status within each group was analyzed using Pearson or Spearman correlation analysis. Additionally, we established a stepwise multivariate linear regression model to assess the influential factors. Collinearity analysis was implemented first. If the variance inflation factor was over 5, the factors would be considered to have multicollinearity and one of them would be excluded from the model based on clinical significance. The stepwise multivariate linear regression analysis was then applied. The inclusion criterion was F ≤ 0.05 and the exclusion criterion was F ≥ 0.1. *P* < 0.05 was considered statistically significant.

## Results

The present study enrolled 32 patients with 64 eyes receiving implantation of ZXR00 IOLs and another 30 patients with 60 eyes receiving monofocal IOLs. The demographic parameters are shown in Table 1.

**Table 1:** Preoperative demographics

Demographic parameter	ZXR00 group	Monofocal group	*P*
Age (years)	68.16 ± 5.44	67.6 ± 7.59	0.921
Sex (male/female)	14/18	9/21	0.263
Axial length (mm)	23.14 ± 0.89	22.72 ± 0.78	0.008*
Corneal astigmatism (D)	0.71 ± 0.40	0.75 ± 0.44	0.732
Corneal endothelial cell count (/mm^2^)	2663 ± 345	2745 ± 319	0.174
IOL power (D)	22.09 ± 2.33	22.88 ± 2.01	0.046*
Target SE (D)	− 0.08 ± 0.12	− 0.08 ± 0.15	0.808

*D* = diopter; *IOL* = intraocular lens; *SD* = standard deviation; *SE* = spherical equivalent *indicates statistical significance

### Static visual acuity

**Fig. 1:**
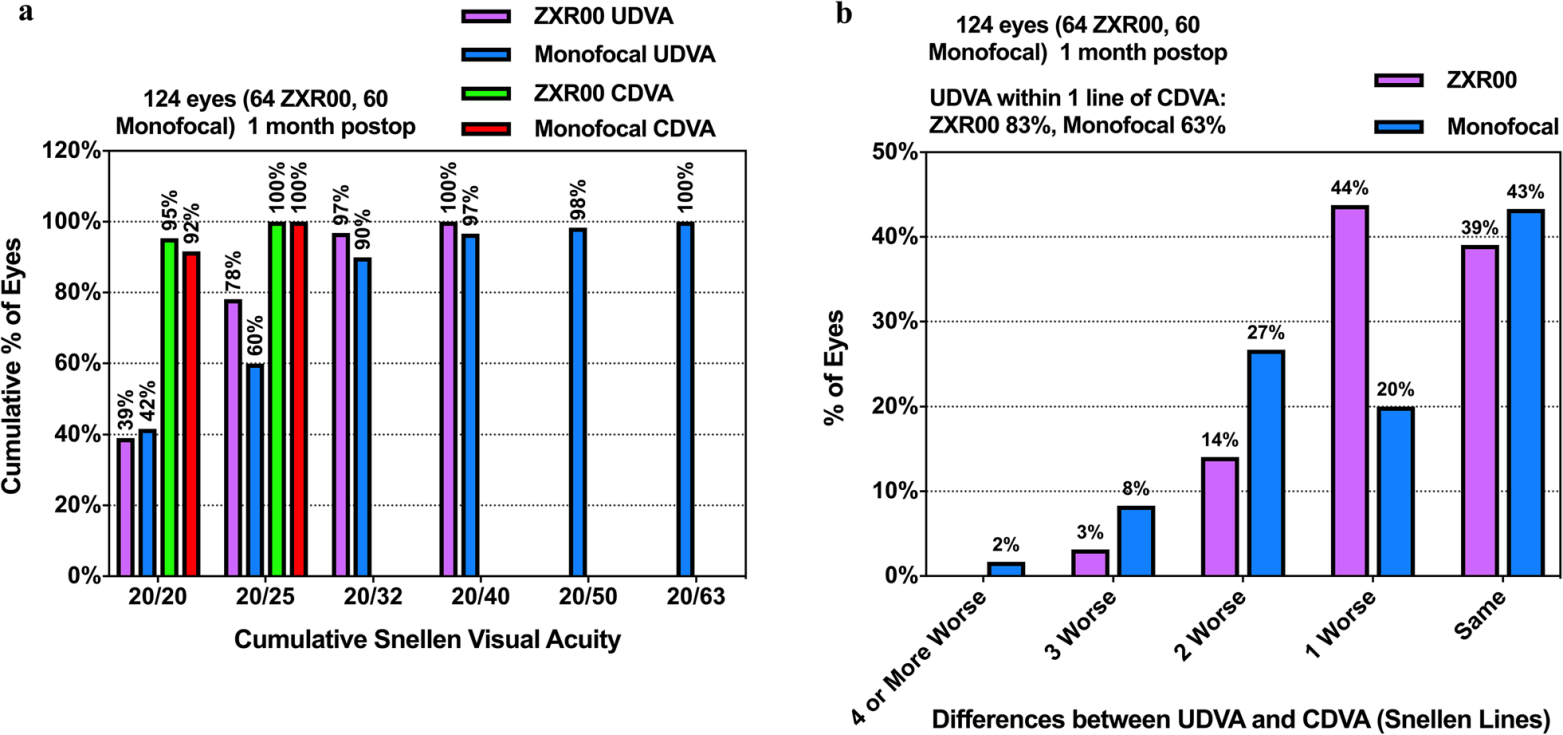
Histograms of the postoperative visual outcomes. **a** Uncorrected and corrected distance visual acuity; **b** Differences between uncorrected and corrected distance visual acuity. CDVA, corrected distance visual acuity; postop, postoperative; UDVA, uncorrected distance visual acuity

The histograms reflecting the efficacy of the surgery are shown in Fig. 1. The results revealed that 78% and 60% in the ZXR00 and monofocal groups achieved 20/25 (Snellen UDVA). The UDVA within one line of CDVA was 83% and 63% in the ZXR00 and monofocal groups. The results of the static VA and the postoperative refraction are summarized in Table 2. The results indicated that the UDVA significantly improved after surgery for both groups (*P* < 0.001). No statistical difference was observed in the postoperative CDVA (*P* = 0.411) and UDVA (*P* = 0.308) between the two groups. The UIVA (*P* = 0.044) and UNVA (*P* = 0.017) were significantly better in the ZXR00 group. Postoperative subject refraction revealed that the ZXR00 group had a more significant myopic deviation (*P* < 0.001). Two groups had similar postoperative diopter of the cylinder (*P* = 0.124).

**Table 2:** Static visual acuity and postoperative refraction

	ZXR00 group (mean ± SD)	Monofocal group (mean ± SD)	*P*
Preoperative			
UDVA (logMAR)	0.383 ± 0.295	0.523 ± 0.375	0.007*
Postoperative			
UDVA (logMAR)	0.086 ± 0.083^#^	0.113 ± 0.119#	0.308
CDVA (logMAR)	0.005 ± 0.021	0.008 ± 0.028	0.411
UIVA (logMAR)	0.280 ± 0.121	0.345 ± 0.169	0.044*
UNVA (logMAR)	0.498 ± 0.147	0.573 ± 0.157	0.017*
Postoperative refraction			
Sphere (D)	− 0.195 ± 0.390	0.275 ± 0.540	< 0.001*
Cylinder (D)	− 0.734 ± 0.415	− 0.596 ± 0.467	0.124
SE (D)	− 0.563 ± 0.404	− 0.056 ± 0.528	< 0.001*

*CDVA* = corrected distance visual acuity; *D* = diopter; *SD* = standard deviation; *SE* = spherical equivalent; *UDVA* = uncorrected distance visual acuity; *UIVA* = uncorrected intermediate visual acuity; *UNVA* = uncorrected near visual acuity

*Indicates statistical significance between groups

^#^Indicates statistical significance *vs.* preoperative visual acuity

### Static and dynamic defocus curve

**Fig. 2:**
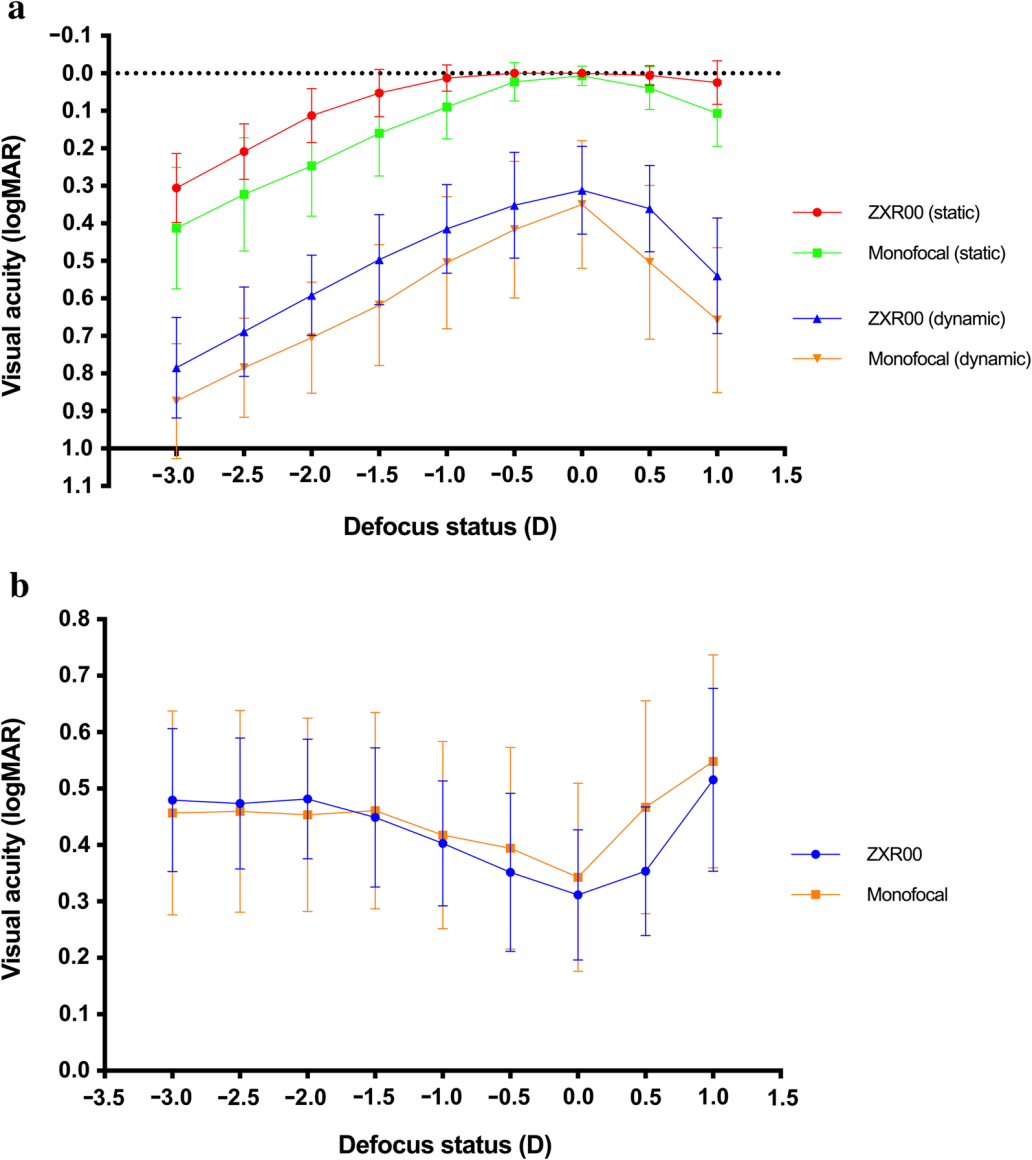
Binocular defocus curve. **a** Binocular static and dynamic defocus curves of the two groups at one month postoperatively. **b** Difference between the dynamic visual acuity and static visual acuity at all defocus statuses at one month postoperatively. D, diopter

The results of binocular static and dynamic VA under different defocus statuses are summarized in Table 3, and static and dynamic defocus curves are shown in Fig. 2a.

**Table 3:** Binocular static and dynamic visual acuity under different defocus statuses

Defocus (D)	Static VA (logMAR, mean ± SD)		*P*	Dynamic VA (logMAR, mean ± SD)		*P*
	ZXR00 group	Monofocal group		ZXR00 group	Monofocal group	
+ 1.0	0.025 ± 0.057	0.107 ± 0.087	< 0.001*	0.540 ± 0.153	0.658 ± 0.192	0.009*
+ 0.5	0.006 ± 0.025	0.040 ± 0.056	0.003*	0.361 ± 0.114	0.504 ± 0.204	0.003*
0.0	0.000 ± 0.000	0.007 ± 0.025	0.141	0.312 ± 0.116	0.350 ± 0.169	0.724
− 0.5	0.000 ± 0.000	0.023 ± 0.050	0.008*	0.352 ± 0.140	0.417 ± 0.181	0.176
− 1.0	0.013 ± 0.034	0.090 ± 0.084	< 0.001*	0.415 ± 0.117	0.505 ± 0.175	0.031*
− 1.5	0.053 ± 0.062	0.160 ± 0.113	< 0.001*	0.497 ± 0.119	0.618 ± 0.160	0.003*
− 2.0	0.113 ± 0.071	0.247 ± 0.133	< 0.001*	0.592 ± 0.106	0.705 ± 0.147	0.002*
− 2.5	0.209 ± 0.073	0.323 ± 0.150	< 0.001*	0.689 ± 0.118	0.785 ± 0.131	0.005*
− 3.0	0.306 ± 0.091	0.413 ± 0.161	0.005*	0.785 ± 0.133	0.874 ± 0.152	0.019*

*D* = diopter; *SD* = standard deviation; *VA* = visual acuity

*Indicates statistical significance

**Fig. 3:**
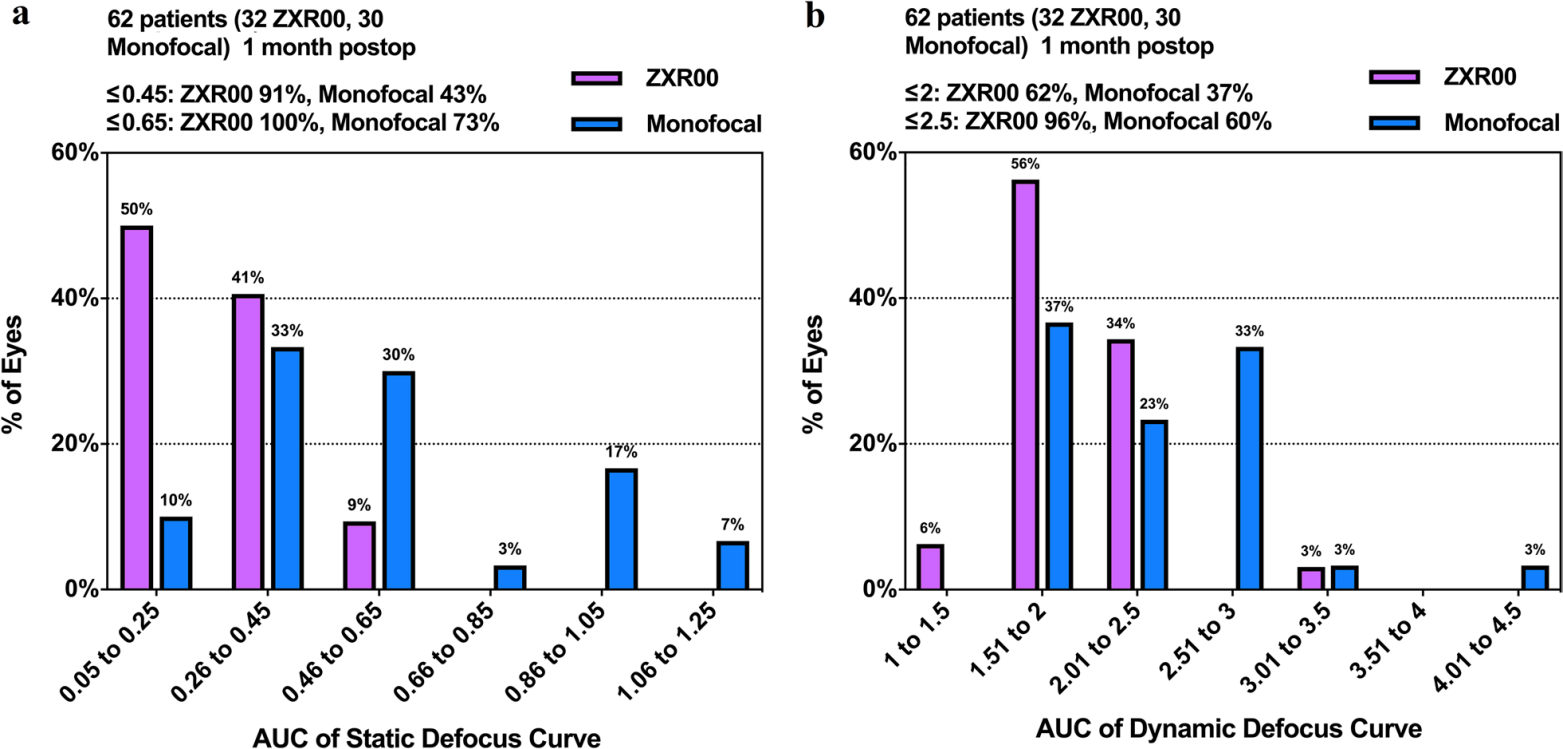
Histograms of the area under the defocus curves. **a** Static defocus curves. **b** Dynamic defocus curves. *AUC,* area under the curve

The static VA in the ZXR00 group was significantly better than that in the monofocal group at all defocus statuses (*P* < 0.05) except 0.0 D (*P* = 0.014). An approximately one-line improvement on the VA chart was observed in the ZXR00 group over the monofocal group from − 1.0 D to − 3.0 D and at + 1.0 D. The binocular static defocus curve revealed a smoother decline from 0.0 D to − 2.0 D in the ZXR00 group compared with the monofocal group. The histograms reflecting the distribution of AUCs are shown in Fig. 3. The AUCs of the static defocus curve were 0.280 ± 0.119 in the ZXR00 group and 0.575 ± 0.262 in the monofocal group. The results indicated that the AUC_static_ of the ZXR00 group was statistically better than that of the monofocal group (*P* < 0.001).

The defocused dynamic VA in the ZXR00 group was significantly better than that in the monofocal group at all defocus statuses (*P* < 0.05) except 0.0 D (*P* = 0.724) and − 0.5 D (*P* = 0.176), and an approximately one-line acuity better was observed. The binocular dynamic defocus curve declined more smoothly from 0.0 D to − 1.5 D in the ZXR00 group. The AUCs and corrected dynamic vision accommodation of the dynamic defocus curve in both groups were calculated. The AUC_dynamic_ and corrected dynamic vision accommodation were 1.939 ± 0.353 D and 5.140 ± 2.175 D in the ZXR00 group, and 2.325 ± 0.559 D and 3.033 ± 2.316 D in the monofocal group. The results showed that the AUC_dynamic_ and corrected dynamic vision accommodation of the ZXR00 group were statistically better than those of the monofocal group (*P* = 0.002 and *P* = 0.001, respectively).

The relationship between the postoperative dynamic and static VA was also analyzed. The postoperative dynamic VA was significantly worse than static VA (*P* < 0.001 in both groups) and showed a significantly positive correlation with static VA (r = 0.979, *P* < 0.001, ZXR00 group; r = 0.951, *P* < 0.001, monofocal group). The differential values between dynamic VA and static VA are demonstrated in Fig. 2b. The curves revealed that the differences became larger with the increase of the defocus diopter from 0.0 D to − 2.0 D in the ZXR00 group (*P* < 0.001) and 0.0 D to − 1.5 D in the monofocal group (*P* < 0.001). There was no statistically significant difference between the two groups regarding the differences between the static and dynamic VA in the defocused statuses (*P* = 0.895).

### Multiple factors analysis on dynamic defocus curve

Stepwise multiple linear regression was performed to fit AUC_dynamic_ and defocused dynamic VA. Age, sex, lens type, corrected dynamic vision accommodation, AUC_static_ and static VA were included in the model. AUC_static_ was excluded from the models fitting the dynamic VA under defocus statuses from − 1.5 D to − 3.0 D as it was collinear with static VA. The results (see Additional file 1) indicated that corrected dynamic vision accommodation was the significant influential factor for AUC_dynamic_ and the defocused dynamic VA (*P* < 0.05) except for − 2.5 D. Defocused dynamic VA was also significantly correlated with static VA at + 1.0 D, + 0.5 D, − 1.0 D, − 1.5 D, − 2.0 D and − 3.0 D (*P* < 0.05). Lens type was a significant factor for dynamic VA at − 2.5 D (*P* = 0.003). AUC_static_ was also a significant factor for AUC_dynamic_ (*P* = 0.034).

## Discussion

The dynamic VA test and static defocus curve test can evaluate the visual function following cataract surgery effectively [7, 14, 15]. Here, we applied a promising method to assess the postoperative all-distance dynamic VA and demonstrated that patients implanted with EDOF IOLs had significantly better overall dynamic defocus curve compared with monofocal IOLs.

The EDOF IOL has an elongated focal zone with the design of multiple concentric diffraction gratings on the optical surface [16]. With this special design, EDOF IOL gains superiority at intermediate performance and acts favorably at distance vision compared with monofocal IOL [16–18]. Our study demonstrated that the ZXR00 IOL provided better UIVA and UNVA compared with the monofocal IOLs. Similarly, the results of the static defocus curve also revealed a better static VA from − 0.5 D to − 3.0 D and a smoother curve from 0.0 D to − 2.0 D for the ZXR00 group. The result is similar to previous studies comparing ZXR00 and monofocal IOLs [5, 19]. Laboratory measurements in an EDOF model eye revealed clearer images at the defocus positions from − 0.75 D to − 1.75 D, and the visualization of the light pathway illustrated elongated focus compared with the distinct single focus in monofocal IOLs [20].

The self-developed dynamic VA evaluation program has been successfully applied to assess the dynamic vision after cataract surgery in our previous studies [14, 15]. The system was further combined with the defocus curve and demonstrated its efficacy in evaluating the dynamic VA under different defocus states [12]. In this study, ZXR00 IOLs provided better defocused dynamic VA from − 1.0 D to − 3.0 D compared with monofocal IOLs. According to the retinal smear theory, moving targets produce marginal artifacts on their retinal images [21]. As the ZXR00 IOLs offer better static VA from − 1.0 D to − 3.0 D, the marginal artifacts for the patients receiving the ZXR00 IOLs are supposed to be slighter than the monofocal IOLs, leading to better dynamic VA. The results of the positive relationship in our study between dynamic and static VA can also support this viewpoint. To evaluate the overall defocus curve, AUC calculation was adopted; it was observed that AUC_dynamic_ was significantly better in the ZXR00 group. The result was consistent with the defocused dynamic VA as the AUC_dynamic_ was an accumulation of each defocused dynamic VA.

The static defocus curve of EDOF IOLs has been well established in previous studies [22–26]. We further evaluated the difference between dynamic and static defocus curves. The difference value increased as the defocus diopter increased in both groups. However, the increase stopped from − 1.5 D in the monofocal group and − 2.0 D in the ZXR00 group. It can be inferred that the EDOF in the ZXR00 IOL retards the descent of the dynamic defocus curve, resulting in the disparity from − 1.5 D to − 2.0 D. The multiple linear regression analysis demonstrated that the corresponding defocused static VA and lens type were the significant influential factors for the defocused dynamic VA from − 1.0 D to − 3.0 D emphasizing the contribution of continuous range of static vision on a more stable dynamic defocus curve. Continuous range of vision is supposed to be a predictive factor of a better defocused dynamic vision.

Corrected dynamic vision accommodation is a new indicator raised in our previous research [12]. As the corrected dynamic VA varies greatly from person to person despite their static VA corrected to 0 logMAR, we put forward this indicator to evaluate the ability to maintain the dynamic VA in defocus statuses, which is similar to the traditional accommodation function calculation. The results demonstrated that the patients with ZXR00 IOL implantation had better corrected dynamic vision accommodation compared with monofocal IOL implantation. It indicated that the ZXR00 IOL provided a more stable dynamic VA as the test distance varied. Additionally, multiple linear regression analysis demonstrated that the corrected dynamic vision accommodation was a crucial influential factor for the defocused dynamic VA rather than static VA, demonstrating its superiority in predicting defocused dynamic VA.

Cataract surgery has gradually become a surgical technique for improving patients’ quality of life. Satisfied static visual function is insufficient for many daily tasks, including sports and driving. Due to the conducting and processing of the dynamic visual signal being different from the static visual signal, dynamic vision evaluation should be performed as a required examination for such a population. Previous dynamic VA tests could only evaluate the ability to identify the moving targets at a certain distance. It is not enough to evaluate the dynamic visual function for patients implanting IOL aiming to achieve all-distance visual function, and the current dynamic defocus curve test could solve the problem. Our new test conveniently assesses dynamic VA at different distances by adding lenses, and thus provides a comprehensive evaluation of all-distance dynamic VA. The dynamic defocus curve test may be widely applicable in the future as an additional measure to the traditional static VA evaluation. The data potentially establishes an IOL selection system based on the dynamic visual function, which is particularly essential for patients with additional requirements for driving and sports. It is noteworthy that the dynamic VA is significantly affected by the degree of refractive error if the refractive error is corrected with glasses [27]. The intrinsic prism and peripheral defocus effect of the glasses might influence the observance of moving targets. Moreover, the visual motion perception is a complicated process affected by neuropsychology and is not fully understood yet [28]. Therefore, the test requires further improvement in the protocol as well as taking the psychological aspects into consideration.

Certain limitations still exist in our study. First, it lacks randomization, and the patients were divided according to their preferences. The preoperative VA also affected the choice of lens type leading to the difference in the preoperative UDVA between the two groups. Second, only one velocity and moving pattern were used in our study to prevent visual fatigue, which is not in accordance with the real world. Hence, additional moving patterns need to be tested. Third, the scheme of the IOL power selection does not include the mini-monovision approach which may improve the overall binocular dynamic visual performance under different defocus statuses. Fourth, the monofocal IOLs are not unified in our study due to the strict health policy on IOL selection. Further studies are still needed to explore the dynamic defocus curve in different types of monofocal and EDOF IOLs. Fifth, the stationary logMAR VA chart used in static defocus curve test may influence the outcomes as a result of memory effects. Gupta’s works [29] pointed out that the combinations of nonrandomized letters and lens presentation should be avoided in defocus curve tests. Computerized static VA chart which can present randomized letters can be used in our future studies.

## Conclusions

In conclusion, the present research demonstrates that EDOF IOL offers equal static distance VA with superior intermediate and near vision compared with monofocal IOL. Analysis of the dynamic defocus curve demonstrates that the overall defocused dynamic vision is better for EDOF IOL including the defocused dynamic VA and corrected dynamic vision accommodation. The corrected dynamic vision accommodation and corresponding static VA are main factors influencing the dynamic visual performance. The dynamic defocus curve test is validated to be an effective method to evaluate continuous dynamic visual quality postoperatively.

## Supplementary Information

**Additional file 1.** Stepwise multiple linear regression analysis for AUC_dynamic_ and dynamic visual acuity in all defocus statuses.

## Data Availability

The data that support the findings of this study are available from the corresponding author upon reasonable request.
