# Supplementary material for: Comparison of dynamic defocus curve on cataract patients implanting extended depth of focus and monofocal intraocular lens
Source: Eye Vis (Lond). 2023 Feb 1;10:5. doi: 10.1186/s40662-022-00323-0 (PMC9890684; doi:10.1186/s40662-022-00323-0)
Supplement: Supplementary file 1 — Additional file 1. Stepwise multiple linear regression analysis for AUCdynamic and dynamic visual acuity in all defocus statuses. [file 40662_2022_323_MOESM1_ESM.docx]

**Additional Information.** Stepwise multiple linear regression analysis for AUC_dynamic_ and dynamic visual acuity in all defocus statuses.

| **Dependent variables** | **Independent variables** | **Standardized coefficient *β*** | ***P*** | **r^2^** |
| --- | --- | --- | --- | --- |
| AUC_dynamic_ | CDVAc | −0.656 | <0.001 | 0.534 |
|  | AUC_static_ | 0.199 | 0.034 |  |
| DVA_+1.0D_ | CDVAc | −0.598 | <0.001 | 0.424 |
|  | SVA_+1.0D_ | 0.201 | 0.047 |  |
| DVA_+0.5D_ | CDVAc | −0.657 | <0.001 | 0.594 |
|  | SVA_+0.5D_ | 0.344 | <0.001 |  |
| DVA_0D_ | CDVAc | −0.498 | <0.001 | 0.235 |
| DVA_−0.5D_ | CDVAc | −0.663 | <0.001 | 0.431 |
| DVA_−1.0D_ | CDVAc | −0.625 | <0.001 | 0.529 |
|  | SVA_−1.0D_ | 0.241 | 0.012 |  |
| DVA_−1.5D_ | CDVAc | −0.565 | <0.001 | 0.422 |
|  | SVA_−1.5D_ | 0.217 | 0.038 |  |
| DVA_−2.0D_ | CDVAc | −0.484 | <0.001 | 0.394 |
|  | SVA_−2.0D_ | 0.298 | 0.006 |  |
| DVA_−2.5D_ | Lens | 0.366 | 0.003 | 0.119 |
| DVA_−3.0D_ | SVA_−3.0D_ | 0.356 | 0.003 | 0.190 |
|  | CDVAc | −0.246 | 0.039 |  |

*AUC* = area under the curve; *CDVAc* = corrected dynamic vision accommodation; *DVA* = dynamic visual acuity; *SVA* = static visual acuity.
